# Complete Genome Sequence of the Hyperthermophilic and Piezophilic Archaeon *Thermococcus barophilus* Ch5, Capable of Growth at the Expense of Hydrogenogenesis from Carbon Monoxide and Formate

**DOI:** 10.1128/genomeA.01534-15

**Published:** 2016-01-14

**Authors:** Philippe Oger, Tatyana G. Sokolova, Darya A. Kozhevnikova, Evgeny A. Taranov, Pauline Vannier, Hyun Sook Lee, Kae Kyoung Kwon, Sung Gyun Kang, Jung-Hyun Lee, Elizaveta A. Bonch-Osmolovskaya, Alexander V. Lebedinsky

**Affiliations:** a Laboratoire de Géologie de Lyon, UMR 5276 CNRS, Ecole Normale Supérieure de Lyon, Lyon, France; b Winogradsky Institute of Microbiology, Research Center of Biotechnology, RAS, Moscow, Russia; c Matís ohf., Food Safety, Environment and Genetics, Reykjavik, Iceland; d Korea Institute of Ocean Science and Technology, Ansan, South Korea

## Abstract

We report here the complete sequence and fully manually curated annotation of the genome of strain Ch5, a new member of the piezophilic hyperthermophilic species *Thermococcus barophilus*.

## GENOME ANNOUNCEMENT

Strain Ch5, a new member of the piezophilic hydrothermal vent archaeal species *Thermococcus barophilus*, has a pressure optimum of 40 MPa. It has been isolated from a deep-sea hydrothermal field of the Mid-Atlantic Ridge (Logachev field chimney, 3,020 m depth) on medium with 0.1 g/liter yeast extract under an atmosphere of 100% CO, on which it grew hydrogenogenically. Strain Ch5 has been assigned to the *T. barophilus* (1) species based on 16S rRNA gene sequence identity (100%). Strain Ch5 can grow organotrophically on yeast extract (0.5 g/liter). Strain Ch5 was among the five *Thermococcus* isolates shown to be capable of formate-driven growth coupled with H_2_ production (2), raising questions as to whether this capacity was determined by genetic determinants that were similar to those identified in *Thermococcus onnurineus* and *Thermococcus gammatolerans*.

The complete genome sequence was determined by a combination of 454 and Illumina sequencing of standard unpaired libraries. *De novo* assembly of the hybrid data with Newbler 2.7 yielded six contigs, which were connected by PCR amplification and Sanger sequencing. The *T. barophilus* Ch5 genome consists of a single circular chromosome of 2,388,527 bp, with an average G+C content of 41.8%. A total of 2,679 protein-coding genes were annotated on the MaGe platform (3–5).

*In silico* DNA-DNA hybridization of the genomes of strain Ch5 and *T. barophilus* MP^T^ confirmed the affiliation of strain Ch5 with the *T. barophilus* species (predicted value, 81.3% ± 2.7% using GGDC 2.0 BLAST+ and the recommended formula 2) (6). However, the genomes differ considerably in size and gene content. The Ch5 genome lacks a plasmid, is ca. 400 kb bigger, and has 310 open reading frames (ORFs) more than the type species genome (7). The *T. barophilus* species core genome is composed of 1,868 families, of which 212 families are specific to *T. barophilus* and absent from other *Thermococcales*. Eighty percent (170/212) of this *T. barophilus*-specific genome encodes conserved proteins of unknown function, while the remainder contains complete loci coding for the degradation of maltose, mannosylglycerate, and threonine. Interestingly, the Ch5 genome contains a chemotaxis locus associated with a flagellum-encoding gene cluster dissimilar from that of strain MP. Major genome rearrangements are associated with putative integrases, transposases, or clustered regularly interspaced short palindromic repeat (CRISPR) loci. Surprisingly, the Ch5 chromosome harbors three *cdc6* homologs, one similar to that of strain MP and two probably resulting from plasmid/chromosome integration events. Strains Ch5 and MP share five highly similar hydrogenase gene clusters, one of which is adjoined by a carbon monoxide dehydrogenase gene and determines the capacity for hydrogenogenic growth on CO. Additionally, strain Ch5 harbors three more hydrogenase gene clusters, one of them encoding a hydrogenase related to F_420_-reducing hydrogenases (8), and two others adjoined by formate dehydrogenase genes. One of these two hydrogenase gene clusters includes a formate transporter gene. This cluster is very similar to those described for *T. onnurineus* and *T. gammatolerans* as genetic determinants of formate-driven growth coupled with H_2_ production (2).

### Nucleotide sequence accession number.

The GenBank accession number of the *T. barophilus* Ch5 genome sequence is CP013050. The version described here is the first version.
